# Sentinel surveillance of COVID-19 in 2024-2025 shows the persistence of Omicron circulation and the introduction of the XFJ recombinant in the Republic of Guinea

**DOI:** 10.11604/pamj.2026.54.6.52267

**Published:** 2026-05-11

**Authors:** Mamadou Bhoye Keita, Bassala Traore, Houssainatou Bah, Mamadou Aliou Sampou, Pépé Tohonamou, Almamy Amara Toure, Moussa Aminata Keita, Mamoudou Conde, Bile Ebi, Sidiki Ibrahima Bamba, Ibrahima Fane, Abdoulaye Diakite, Abdoulaye Fodé Toure, Sékou Oumar Traore, Mamadou Dian Djiwo Diallo, Oumou Salamata Diallo, Mamadou Baghirou Bah, Mafoudia Soumare, Kaba Kourouma, Pierre Fenano, Mahamoud Sama Cherif

**Affiliations:** 1 National Public Health Institute, Conakry, Republic of Guinea,; 2 Gamal Abdel Nasser University of Conakry, Conakry, Republic of Guinea,; 3 N'Zérékoré University, N'Zérékoré, Republic of Guinea,; 4 Department of Public Health, Faculty of Health Sciences and Techniques, Gamal Abdel Nasser University, Conakry, Republic of Guinea,; 5 FHI360, Regional Office for West Africa and the Middle East/Guinea, Conakry, Republic of Guinea

**Keywords:** Guinea, sentinel surveillance, COVID-19, variant, recombinant XFJ

## Abstract

**Introduction:**

several studies have shown the evolution of SARS-CoV-2 between 2020 and 2023 in Guinea. This study provides updated information on the circulation dynamics of SARS-CoV-2 from 2024 to 2025 and the variants circulating in Guinea.

**Methods:**

as part of the integrated influenza/COVID-19 sentinel surveillance program, 2423 nasopharyngeal samples were analysed using RT-PCR, and those positive for SARS-CoV-2 were sequenced using the Illumina COVIDSeq Assay protocol.

**Results:**

all age groups, ranging from <2 years to > 64 years, were affected by COVID-19, with an average SARS-CoV-2 positivity rate of 2.3%. The virus showed sporadic activity throughout the 2024-2025 period, with a higher level of activity during epidemiological week 42 (October) of 2024. Genomic analysis showed that only the Omicron variant circulated, along with its subvariants JN.1 and LF.7, in 2024. Subvariant LF.7 and recombinant XFJ also circulated in 2025.

**Conclusion:**

SARS-CoV-2 subvariants continue to circulate sporadically in Guinea, with the XFJ recombinant having been detected in humans. Therefore, it is imperative to strengthen sentinel, genomic, and environmental surveillance for the early detection of any new COVID-19 outbreak and the potential emergence of new variants.

## Introduction

COVID-19 triggered one of the deadliest pandemics in human history, causing over 6 million deaths worldwide [1-3]. Several coronaviruses are responsible for respiratory infections, but the infections themselves usually remain mild. SARS-CoV-2 distinguishes itself from the others through its high transmissibility and infection rates [4]. There was a spike in the activity of the COVID-19 pandemic from late 2019 until early 2023, before the World Health Organization (WHO) declared the pandemic was no longer a global health emergency on May 4, 2023, marking its end [5].

Although it is no longer a global health emergency, it continues to claim victims, with over 400,000 new cases and over 3,000 deaths each week worldwide. By May 2023, over 766 million SARS-CoV-2 infection cases had been reported, resulting in over 6.9 million deaths [6]. The WHO´s announcement that we are now in the post-pandemic period does not translate into the end of the COVID-19 global health burden. Health systems must have the capacity to predict virus circulation periods to improve information dissemination and adoption of non-pharmaceutical measures to break the chain of transmission during these periods [7]. By December 2024, 776,897,200 positive cases had been recorded, resulting in 7,076,329 deaths [6]. Africa´s first recorded COVID-19 case was detected in Egypt in returning travellers on 14 February 2020 (9) [8]. The WHO African Region had recorded over 9,538,679 confirmed cases and over 175,394 deaths by June 28, 2023 [9].

COVID-19 had a major impact on the circulation of other respiratory pathogens due to non-pharmaceutical measures, leading to low circulation for some pathogens and extinction for others. However, SARS-CoV-2 continues to circulate alongside other respiratory pathogens, as reported in several studies conducted in Africa and worldwide. This co-circulation or co-detection poses new clinical and microbiological challenges. Due to this simultaneous circulation, the disease can progress to more severe forms, leading to a high number of hospitalisations and a need for artificial or mechanical respiratory assistance. These phenomena are associated with a significantly elevated mortality risk [10-14]. According to the WHO, Guinea has recorded approximately 38,582 positive cases, including 468 deaths, and a vaccination coverage of 47% among the elderly since health authorities announced the first case on 12 March 2020 [15,16].

Efforts have been made in Guinea to acquire further insight into COVID-19, particularly with respect to its epidemiology, evolutionary dynamics, genomic sequences, and variants circulating from March 2020 to 2023 [17-19]. However, since 2024, there has been a lack of data concerning SARS-CoV-2 circulation in Guinea. Thus, in this study, we aimed to provide an update on the epidemiological and virological dynamics of SARS-CoV-2 from 2024 to 2025. It is important to recall that genomic surveillance of this pathogen remains essential not only for the early detection of the potential emergence or re-emergence of variants but also for better understanding its evolution, anticipating health risks, and strengthening response capacities against potential future crises.

## Methods

**This study is part of an integrated Influenza and COVID-19 surveillance program conducted in Guinea from 2024 to 2025:** this was a cross-sectional study conducted from 2024 to 2025. The nasopharyngeal samples were collected at ten sentinel sites, with eight located in Conakry and two lying on the periphery of Conakry. The sentinel sites consisted of four (4) influenza-like illness (ILI) sites: the Kouléwondy health centre in Kaloum commune, the Maciré health centre in Dixinn commune, the Gbèssia port 1 health centre in Gbèssia commune, and the Km 36 health centre in Sanoyah commune. The six (6) severe acute respiratory infection (SARI) sites were the Ignace Deen University Hospital (CHU) in Kaloum commune, the Donka University Hospital (CHU) in Dixinn commune, the Ratoma communal medical centre (CMC) in Ratoma commune, the Tinan Guinée clinic in Ratoma commune, the Mafèrinyah improved health centre (CSA) in the urban commune of Forécariah, and the Dubréka prefectural hospital in the urban commune of Dubréka.

**Population and sample collection:** patients suspected of having ILI or SARI were selected according to the WHO case definitions [20,21]. Patients with incomplete data or inadequate samples were excluded from the analysis. A total of 2423 patients were enrolled during the study period. Nasopharyngeal or oropharyngeal swabs were obtained from each patient. Each swab was placed in a viral transport medium (VTM) and transported to the National Public Health Institute (INSP) for analysis.

**Nucleic acid extraction and amplification:** we used a manual extraction technique to extract nucleic acid from 200µl of samples using the Qiagen QIAmp viral RNA kit according to the manufacturer's recommendations. RNA was then eluted in 100µl of elution buffer provided with the kit. The eluted RNA was used to amplify the target sequence of SARS-CoV-2 (the ORF 1a/b gene and the nucleocapsid N gene) with the Sansure Biotech Novel Coronavirus (2019-nCoV) Nucleic Acid Diagnostic Kit (PCR-Fluorescence Probing) via an ABI 7500 Fast Dx Real-time PCR machine (Applied Biosystems, USA) under the following PCR conditions: reverse transcription (50°C for 30 min, 1 cycle), cDNA pre-denaturation (95°C for 1 min, 1 cycle), denaturation (95°C for 15 sec, 45 cycles), hybridisation, elongation, and fluorescence collection (60°C for 31 sec, 45 cycles). The human ribonuclease P gene was amplified for each sample to assess its integrity, and it was also used as an internal control. The data generated after amplification were analyzed using ABI Sequence Detection Software version 1.4 (applied biosystems).

**Sequencing:** we sequenced fifteen (15) samples, i.e., 26.31% of positive cases with a Ct < 28. We used Illumina technology for the whole-genome sequencing of SARS-CoV-2. Complementary DNA (cDNA) was synthesized from the extracted RNA using the Illumina cDNA synthesis kit (USA). We then amplified it using the Illumina COVIDSeq Assay Box 3 kit (USA) containing SARS-CoV-2-specific primers. After amplification, the PCR product was fragmented and tagged using Illumina DNA prep PCR + Buffer and Illumina COVIDSeq Assay Box 2 kits (USA). Tagmentation was then stopped, and washing was performed using the Illumina DNA prep IPB+Buffer kit (USA). The tagged DNA was then amplified with adapter indices using Illumina DNA prep PCR+Buffer and Illumina index set 1 kits (USA). The library was pooled and washed to remove adapter indices not bound to the DNA with the Illumina COVIDSeq Assay Box 1 kit (USA). The washed library was quantified, normalised, and loaded onto an Illumina iSeq100 platform according to the manufacturer's recommendations. After sequencing, the FASTQ format data generated by the sequencer were retrieved and analysed using the online CZ-ID platform [22], with default parameters for sequence quality control, base calling, and consensus generation. We then used the FASTA files to generate a temporal phylogenetic tree using Nextstrain. The sequences were deposited in the GenBank database under the following accession numbers: sublineage JN.1 (GenBank accession number PX491151), sublineage LF.7 (GenBank accession number PX486808), and recombinant XFJ (GenBank accession number PX491150).

**Data analysis:** the socio-demographic and clinical data on the patients were entered into Excel 2016 software and then exported to R software version 4.1.0 for analysis. We determined the proportion of sociodemographic characteristics (sex, age, and sentinel site) based on the total number of enrolled patients. For confirmed cases, the positivity rate was calculated for each age group relative to the total number of positive cases. The difference between the categories was examined using the Chi-square independence test. To observe viral circulation, we linked positive cases to different epidemiological weeks and months.

**Ethical considerations:** by the Republic of Guinea law, this study did not require approval by the health research ethics committee because it was an observational study for disease surveillance. The surveillance protocol was approved by the Ministry of Health and Hygiene as part of the surveillance of diseases with epidemic potential.

## Results

A total of 2423 samples were analysed to detect SARS-CoV-2. The results obtained after amplification of the extracted RNA revealed the presence of SARS-CoV-2 target sequences in 57 patients, with an overall positivity rate of 2.4% and a 95% CI (1.8-3.1).

**Sociodemographic characteristics:** the results of this study show that the 15-50-year-old age group was the most represented, at 810 cases (33%), with a 95% CI of 32-35, followed by the <2-year-olds group, at 776 cases (32%), with a 95% CI of 30-34. The least represented age group was 50-64 years of age, at 144 cases (6.8%), with a 95% CI of 5.1-7.0. Males were the most represented in this study, with 1302 cases (54%) and a 95% CI of 52-56, while for females, there were 1121 (46%) cases, with a 95% CI of 44-48. The Gbèssia Port 1 health centre was the most represented site, with 445 cases (18%) and a 95% CI of 17-20, followed by the Pneumology Department of the Ignace Deen National Hospital, with 416 cases (17%) and a 95% CI of 16-19. The least represented site was the Dubréka prefectural hospital, with 78 cases (3.2%) and a 95% CI of 2.6-4.0 (Table 1).

**Table 1:** proportions of COVID-19-positive patients arranged according to sociodemographic characteristics

Characteristics	Total; N (%)	Negative; N (%)	Positive; N (%)	P-value
**Age**				**0.026**
<2	776 (32%)	766	10 (1.3%)	
2-4	233 (9.6%)	229	4 (1.7%)	
5-14	296 (12%)	290	6 (2.0%)	
15-50	810 (33%)	785	25 (3.1%)	
50-64	144 (5.9%)	141	3 (2.1%)	
≥65	164 (6.8%)	155	9 (5.5%)	
**Sex**				**0.3**
Female	1121 (46%)	1091	30 (53%)	
Male	1302 (54%)	1275	27 (47%)	
**Site**				
Gbèssia Port 1 Health Centre	433 (18%)	439	6 (11%)	
Km36 Sanoyah Health Centre	321 (13%)	313	8 (14%)	
Kouléwondy Health Centre	331 (14%)	317	14 (25%)	
Maciré Health Centre	340 (14%)	333	7 (12%)	
Tina Guinée Clinic	108 (4.5%)	105	3 (5.3%)	
Ratoma Municipal Medical Center	152 (6.3%)	148	4 (7.0%)	
Maférinyah Improved Health Center	139 (5.7%)	138	1 (1.8%)	
The Dubréka Prefectural Hospital	78 (3.2%)	76	2 (3.5%)	
Donka Pediatrics Department	93 (3.8%)	93	0 (0%)	
Ignace Deen Pneumology	416 (17%)	404	12 (21%)	

The results show that 15-50-year-olds were the most affected, accounting for 25 (44%) of all positive cases, followed by the < 2 years group, accounting for 10 (18%). The age groups least affected by SARS-CoV-2 were 50-64-year-olds, with three cases (5.3%), and 2-4-year-olds, with four (7.0%), with a p = 0.026. The positivity rates among women and men were close, at 53% and 47%, respectively. The Kouléwondy health centre recorded the most positive cases, with 14 (25%), followed by the Pneumology Department of Ignace Deen University Hospital, at 12 (21%). The sites with the fewest positive cases were the Maférinyah improved health centre, with one case (1.8%), and Dubréka prefectural hospital and Maciré health centre, with two (3.5%) and seven (12%) cases, respectively. No positive cases were detected in the paediatrics department of Donka University Hospital (Table 1).

**Figure 1:**
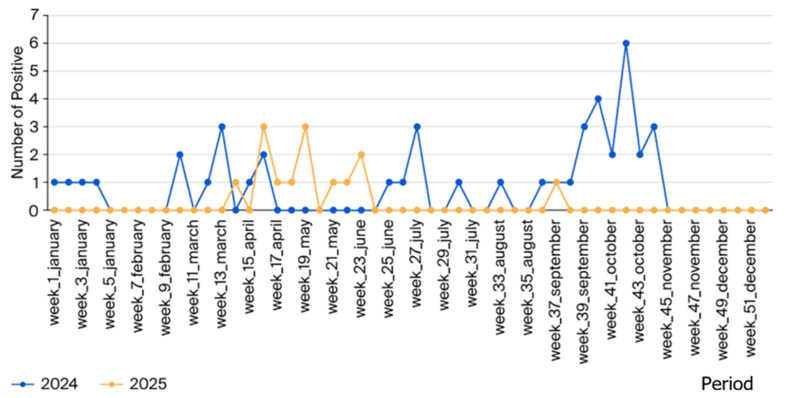
distribution of SARS-CoV-2 according to epidemiological weeks; each colour represents an epidemiological year

We monitored the dynamics of SARS-CoV-2 over the 2024-2025 period to reveal the virus´s periods of activity even after the declaration of the end of the COVID-19 pandemic. We observed sporadic circulation at the beginning of 2024 (weeks 1-4), with a low detection rate (1 sample per week), followed by disappearance throughout February. From March (week 10), a resumption of virus activity was noted, with a small peak at week 13 (three samples). This activity continued until week 17 (April) before disappearing again. No viral activity was observed from the end of April (week 17) until the end of the second week of June (week 25). Sporadic activity resumed starting this week, with a new peak in week 27 (July). Only one positive case was detected throughout August (week 33). Strong virus activity was observed from week 36 (September) and persisted throughout September, with a significant peak at week 42 in October (six samples), followed by disappearance at week 44 until the end of the year. In 2025, the virus exhibited sporadic activity. Unlike in 2024, no activity was observed at the start of the year. The first positive case was detected in April (Week 14). This was followed by several other cases in the following weeks, with two peaks at week 16 (April) and week 19 (May). This activity continued until week 23 (June) before disappearing in week 24. No activity was observed during the following two months; activity reappeared in September in week 37 (Figure 1).

**Figure 2:**
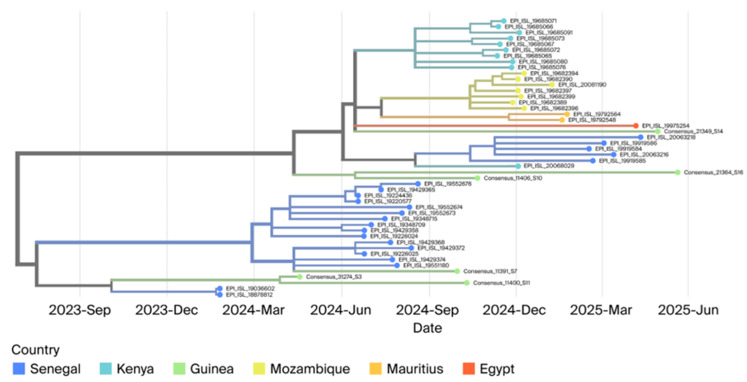
temporal phylogenetic tree of SARS-CoV-2 sequences; branches are coloured according to the sequences´ countries of origin

**Sequencing and bioinformatics analysis:** all fifteen sequenced samples corresponded to the Omicron variant. We found twelve samples of the 24A subvariant (sublineage JN.1). We deposited the whole-genome sequences of two samples with the best coverage (86.06% and 95.06%) in GenBank (accession numbers PX480764 and PX491151). We also found two samples of the 24H subvariant (sublineage LF.7), with whole-genome sequence coverages of 92.15% and 91.41% (accession numbers 486808 and PX491146). We also found another whole-genome sequence with 92.86% coverage, which was an XFJ recombinant (accession number PX491150) (Figure 2).

**Figure 3:**
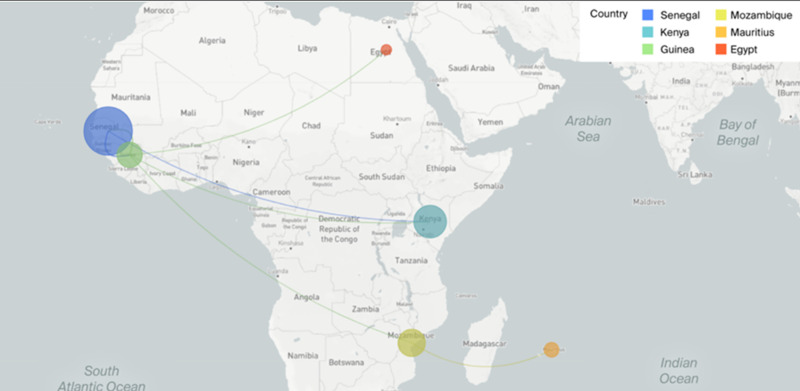
transmission and geographical circulation of SARS-CoV-2 sequences; each circle represents a country, and its size is proportional to the number of sequences present; arrows indicate the inferred transmissions

To better compare our sequences with those from Africa, we searched for several SARS-CoV-2 sequences deposited by countries such as Senegal, Mozambique, Egypt, Kenya, and Mauritius in the GISAID database, allowing us to assess the evolutionary dynamics of SARS-CoV-2 as well as the possible introduction of variants in the Pan-African context. The map below (Figure 3) illustrates the possibility of transmission between countries, highlighting the main flows from Senegal

The similarity of these sequences with those from Senegal and other neighbouring regions suggests that transmission is both community-based and linked to cross-border movement. These data confirm the importance of maintaining active genomic surveillance to detect the emergence of new variants and better understand transmission chains in order to guide virus prevention and control strategies in Guinea.

## Discussion

The first case of COVID-19 in Guinea was detected on 19 March 2020 at the National Public Health Institute Laboratory owing to its experience with influenza surveillance [23]. Subsequently, seven laboratories were equipped with diagnostic capacity in the country, and rapid antigen tests were evaluated and introduced in July 2021. Currently, three laboratories participate in genomic surveillance to identify the different SARS-CoV-2 variants circulating in Guinea. Thus, the variants that circulated in Guinea from 2020 to 2023 have been well described: Alpha, Delta, Eta, and Omicron, along with their subvariants [16-19]. Therefore, the objective of this study was to provide an update on variant circulation data in Guinea, particularly from 2024 to 2025, as well as the epidemiological profiles of patients. The INSP has been conducting integrated influenza and COVID-19 surveillance since 2022 [24]. To perform this study, we involved all sentinel sites of the integrated influenza and COVID-19 surveillance system in Guinea. These surveillance data show that SARS-CoV-2 continues to circulate sporadically in Guinea, and Omicron subvariants have been identified.

The positivity rate continues to decrease over time, as it has from 2020 to 2025, and ranges between 22.30% and 2.3% [17]. This can be explained by the acquisition of mass immunity against SARS-CoV-2 due to vaccination and/or human contact with the virus [25]. Nevertheless, surveillance must be maintained to detect the emergence of any new variants that could overcome herd immunity. Furthermore, these data show that all age groups are affected, although children and the elderly are the groups most vulnerable to COVID-19 and influenza in Guinea, as shown in Table 1 [26]. Regarding the periodicity of circulation, we observed sporadic activity of SARS-CoV-2 throughout 2024-2025. Additionally, peaks were observed at the beginning of the dry season in 2024 (October) and at the beginning of the rainy season in 2025 (May). It should also be noted that during the 2024-2025 period, only Omicron subvariants circulated in Guinea.

In 2024, subvariants 24A (sublineage JN.1) and 24H (sublineage LF.7) were observed. These two subvariants had been reported in the previous 2023-2024 surveillance data (19). In 2025, subvariant 24/LF.7 continued to circulate, and we also recorded the introduction of recombinant XFJ into the country. The source of this newly introduced XFJ recombinant in Guinea could be the Arab Republic of Egypt (Figure 3). Notably, the XFJ recombinant results from recombination between sublineage JN.1 (BA.2.86.1.1) and subvariant XBB.1.5 (clade 23A), both of which also belong to the Omicron variant and are already circulating in Guinea [17].

## Conclusion

In summary, this study allowed us to understand the dynamics of SARS-CoV-2 in Guinea from 2024 to 2025. Omicron subvariants detected since 2023 continue to circulate sporadically, and the XFJ recombinant was detected for the first time in humans in Guinea. All age groups and study areas in Conakry and the surrounding prefectures were affected by respiratory infections due to SARS-CoV-2. Therefore, it is imperative to strengthen genomic and environmental surveillance to enable early detection of variants in order to prevent and effectively respond to any new COVID-19 outbreak.

### What is known about this topic

- Since the beginning of the SARS-CoV-2 pandemic in 2020, several variants have circulated in Guinea until 2023; no published information is available on the evolution of this virus in Guinea.

### What this study adds

- An update on the information regarding the dynamics of SARS-CoV-2 circulation from 2024; the introduction of an XFJ recombinant of the Omicron variant.
